# The impact of urban areas on the water quality gradient along a lowland river

**DOI:** 10.1007/s10661-016-5638-z

**Published:** 2016-10-18

**Authors:** Katarzyna Glińska-Lewczuk, Iwona Gołaś, Józef Koc, Anna Gotkowska-Płachta, Monika Harnisz, Andrzej Rochwerger

**Affiliations:** 1 Department of Environmental Microbiology, University of Warmia and Mazury in Olsztyn, Prawocheńskiego 1, 10-759 Olsztyn, Poland; 2 Department of Water Resources, Climatology, and Environmental Management, University of Warmia and Mazury in Olsztyn, Plac Lodzki 2, 10-759 Olsztyn, Poland

**Keywords:** Lowland river, Urban area, Water quality, Microbiological pollution

## Abstract

The effects of five towns on river water pollution were examined along the Łyna River (southern watershed of the Baltic Sea, northern Poland). The relationships among the spatially derived indicators of urbanization, environmental variables, and physico-chemical and microbiological data (heterotrophic plate count at 22 and 37 °C, and fecal coli) obtained from longitudinal river profiling have been examined with the use of multivariate analyses such as principal component analysis with factor analysis (PCA/FA) and hierarchical cluster analysis (HCA). We recognized the river channel as an environmental path that links serial urban areas into an “urban river continuum.” An overall increasing trend in nutrients and indicator bacteria from suburban headwaters to urbanized sections of the river was detected despite a significant decrease in those between the towns. We concluded that the role of a multicity is equally as important as a single urban area in predicting the impacts of man-made pollutants on river water quality.

## Introduction

Rivers, being a natural interface between watersheds and the seas, are a medium that transports both substances of natural origin and pollutant loads from anthropogenic sources. Among various human activities within their watersheds, urban areas are reported to have the most consistent and ubiquitous effects on surface water quality (Potasznik and Szymczyk 2015; Mallin et al. 2016), habitat alteration (Hatt et al. 2004; Elmore and Kaushal 2008), and reduction in biodiversity (Spänhoff et al. 2007; Obolewski 2013) due to both the significant load of pollutants from point and non-point sources and the increased impervious surface cover. Direct runoff from urbanized surfaces and sewage discharges not connected to a wastewater treatment plant (WWTP) has emerged as a serious threat, not only to the ecological values of water ecosystems but also to the provision of good quality water required for all socio-economic functions (Kaushal and Belt 2012; Brion et al. 2015; Gotkowska-Płachta et al. 2016). The observations of ecological degradation of streams draining urban lands enabled Walsh et al. (2005) to formulate the term “urban stream syndrome,” which means negative changes in flow regime, riverbed morphology, and increase in pollutants’ loads. Although the mechanisms driving the syndrome are complex, the most significant impacts reported in the literature refer primarily to the imperviousness of the total catchment area (Walsh et al. 2012) and stormwater runoff delivered to streams by urban drainage systems (Kaushal and Belt 2012). In light of these factors, the considerable monitoring regarding the seasonal variation of environmental factors (temperature, precipitation, and flows) on the physico-chemical and microbiological properties of urban streams has been reported (Brooks et al. 2006, Schoonover et al. 2006, Walsh et al. 2016).

Although the impact of urban pollution on aquatic ecosystems has been well described in the literature (e.g., Servais et al. 2007; Daly et al. 2013; Garcıa-Armisen et al. 2014; Begum et al. 2016), few studies regarding the significance of consecutive towns located along one river on water pollution have been carried out. In the present study, we monitored the changes in water quality parameters in a lowland river passing through five towns with a dynamic conceptual framework for investigating important research questions: (i) how do the water quality parameters, including nutrients and bacteria, change in space and time under environmental site-specific factors such as flow rate, precipitation, and anthropogenic variables due to the input of wastewater or population size connected to WWTPs?; (ii) what is the gradient of river pollution along the river course passing through the urban areas?; and (iii) what are the overlapping (cumulative) effects of urban pollution on water quality in the receiving river or the potential for self-purification downstream of the urban areas? We hypothesized that the river water pollution is related to the anthropogenic activities along the river and that consecutive towns may have a cumulative or overlapping effect on the river’s functions. Based on hydrochemical and microbiological profiling along the river course, we applied data reduction techniques as principal component analysis and factor analysis (PCA/FA) to evaluate the importance of various water quality parameters and determine the most meaningful parameters responsible for the spatial and temporal variations in river water quality. Finally, the objective of this study was to quantify the seasonal and spatial distribution of common indicator bacteria, including heterotrophic psychrophilic bacteria (HPC 22), heterotrophic mesophilic bacteria (HPC 37), and fecal coliforms (FC), along a lowland postglacial river located in the temperate climate zone under the pressure of urban development.

## Methods

### Site description

**Fig. 1:**
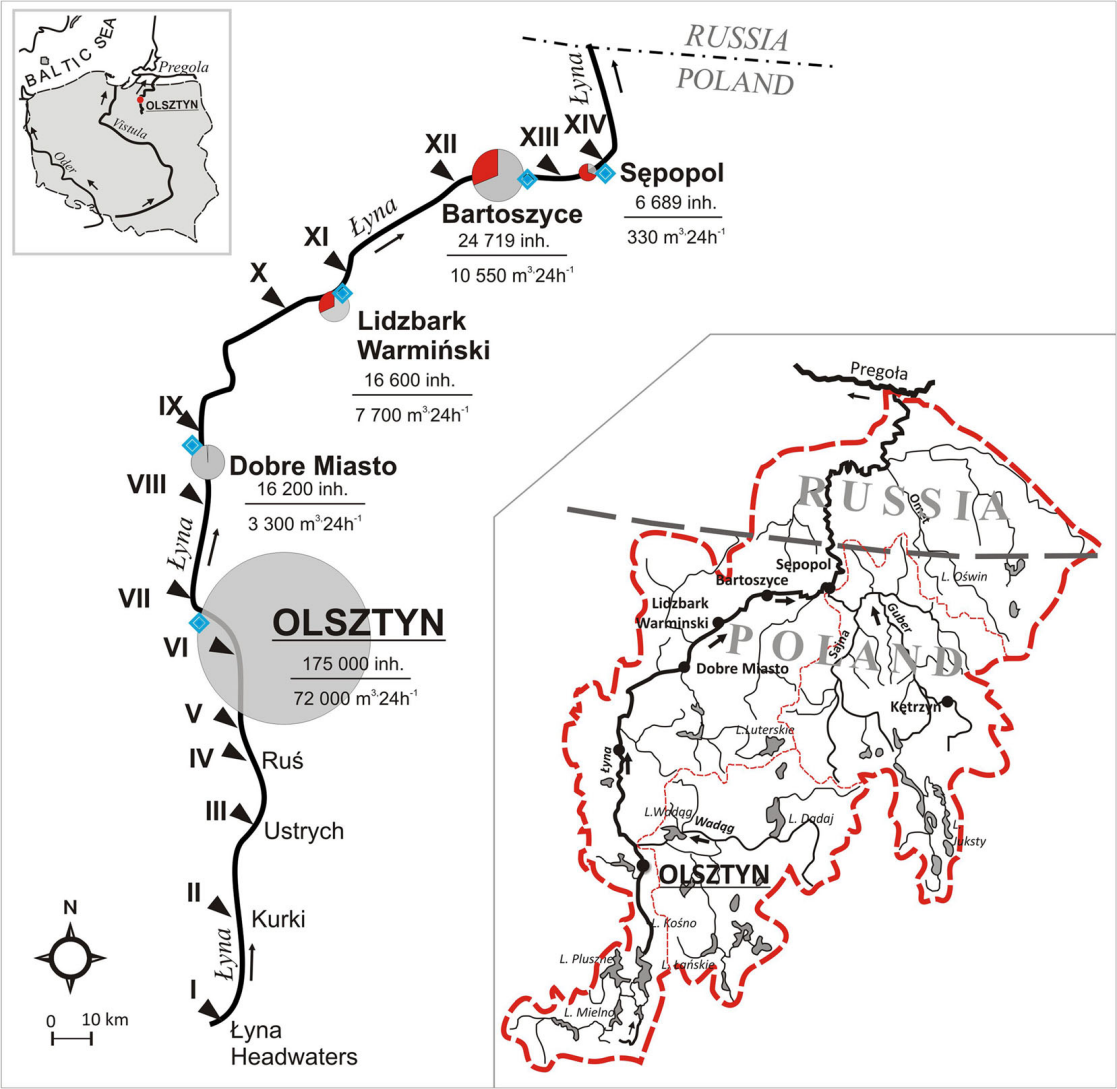
Location of the study area on the background of the Łyna River catchment. Sampling stations (*I*–*XIV*) are marked along the Łyna River with *black triangles*. The *size of a circle* is related to the population in the town. *Gray color* in the *circle* denotes inhabitants served by the wastewater treatment plants (WWTP), whereas *red color* denotes a share of inhabitants not served by the WWTP. *Blue diamond symbol* denotes WWTP placement. The *upper number* in a fraction denotes the population in the town, while the *lower number* is the daily flow rate of the WWTP

River corridors in northeastern Poland (region called the “Green Lungs of Poland”) are widely perceived to be of high ecological quality, with flow regimes typical of temperate climate zones in the Middle European lowlands. The Łyna River is the main watercourse in the region in terms of the total catchment area (7126 km^2^) and total length (264 km) (Fig. 1). The river originates from a headwater area (263 km from the river mouth) at a height of 155 m a.s.l. This section of the river shows outstanding natural values with limited human activity. The catchment is located in postglacial area with mosaics of soils, with feature characteristic of morainic hills used by agriculture (53 %), forests (29 %), lakes (7 %), and built-up areas (11 %) (Glińska-Lewczuk and Burandt 2011). The agricultural areas in the region are used by private farmers. Soils are typically fertilized with N inorganic salts (KNO_3_) with a load of 110–124 kg NPK ha^−1^ year^−1^, including N as 75.1 ha^−1^ year^−1^ (GUS 2012). The high retention indices of its catchment result from the numerous lakes, prevailing sharing of permeable soils, and dense forest cover, which attenuates any flood wave in its upper course (Glińska-Lewczuk 2006).

The average river flow at the gauge in Sępopol (Polish–Russian border) in the study period (November 2011–October 2012) was 23.4 m^3^ s^−1^, ranging from 13.3 m^3^ s^−1^ during the summer low water period to 62.8 m^3^ s^−1^ during spring floods. The runoff per unit area is generally low (5–7 L km^−2^ s^−1^) due to moderate rainfall totals (620 mm on average) and high evapotranspiration values (570 mm). According to the Institute for Meteorology and Water Management in Poland (IMWM), the study period (2012) cover the wet year with rainfall sums which exceeded the multiyear average and amounted to 738 mm in Olsztyn and 783 mm in Lidzbark Warmiński.

At a distance of ca. 45 km from the springs, the Łyna River reaches Olsztyn—the first of the five towns on its way. The total urban area (towns on the Łyna River) covers 122.74 km^2^, which is 2.15 % of its catchment within the Polish border. The town of Olsztyn is the most populated area (175,000 inhabitants) and the biggest (87.9 km^2^) in the region. Downstream of Olsztyn, the Łyna River receives effluents from a WWTP with a capacity of 72,000 m^3^·24 h^−1^. Downstream the river, main sources of pollutants are WWTP effluents from the towns of Dobre Miasto (16,200 inhabitants), Lidzbark Warmiński (16,600 inhabitants), Bartoszyce (24,700 inhabitants), and Sępopol (6689 inhabitants). Each of these towns has its own WWTP. Each of the WTTPs treats wastewater to the secondary level but none of them applies disinfection, as it is not compulsory according to the Polish standards (Journal of Laws of 2006, no. 137, item 984). The share of the population connected to the sanitary network ranges from 68 % for Dobre Miasto to 100 % for Olsztyn. The remaining population uses septic systems or a home sewage treatment plants.

### Water sampling and analytical procedure

The present study was conducted from November 2011 to October 2012 along a 198-km section of the Łyna River within the Polish border. Water sampling was carried out on a bimonthly basis from 14 sampling stations (Fig. 1; Table 1) established along both natural (upstream Olsztyn) and urban (downstream Olsztyn) sections of the river. The sampling design aimed to reflect the spatial and seasonal variations in the chemical composition of water, with special emphasis on the effects of urban areas on the river’s health. All samples were taken upstream of each town and downstream, ca. 200 m beneath the WWTP effluents, to avoid influence from the river tributaries.

**Table 1:** List of sampling sites along the Łyna River and their characteristics

Station no.	Sampling station on the Łyna River	Catchment area A (km^2^)	Distance from river mouth L	Location above sea level	Population (no. of inhabitants)	River flow Q (m^3^ s^−1^)		Daily mean wastewater flow	Sewered population
			(km)	m a.s.l.		Min–max	Mean	m^3^·24 h^−1^	%
I	Headwaters	5.8	263.1	150.0	50	0.1–0.2	0.1		
II	Kurki	358.6	250.1	135.2	100	1.1–3.4	2.2		
III	Ustrych	437.7	237.0	133.2	50	1.8–3.8	2.8		
IV	Ruś	474.4	220.7	126.2	400	2.8–4.2	3.2		
V	Upstream of Olsztyn	567.7	218.7	114.5	1200	2.8–5.4	3.7		
VI	Olsztyn center	578.6	213.5	109.5	175,000	3.1–4.8	4.1		
VII	Downstream of Olsztyn	1786.4	212.5	104.2		7.1–13.1	8.9	72,000	100.0
VIII	Upstream of Dobre Miasto	2034.9	173.9	79.1	450	8.2–21.9	13.1		
IX	Downstream of Dobre Miasto	2056.8	167.3	74.9	16,200	9.6–22.4	13.8	3300	69.8
X	Upstream of Lidzbark Warmiński	2426.1	127.9	70.0	600	12.1–20.6	15.9		
XI	Downstream of Lidzbark Warmiński	2449.3	125.3	66.3	16,600	12.2–25.7	20.5	7700	78.2
XII	Upstream of Bartoszyce	3169.6	94.3	57.6	1000	12.4–30.9	21.6		
XIII	Downstream of Bartoszyce = upstream of Sępopol	3606.1	72.1	32.1	24,719	12.9–39.9	22.8	10,550	99.8
XIV	Downstream of Sępopol	5314.9	63.9	25.0	6689	13.3–62.8	23.4	330	84.5

Water temperature (°C), dissolved oxygen (DO, mg L^−1^), and pH were recorded in situ using the calibrated multiparameter probe YSI Professional Plus, USA. The water samples were collected in 5-L sterile polyethylene bottles and kept cold in the dark until they were analyzed within 12 h. The laboratory analyses of the water included the following nutrients: nitrate nitrogen (NO_3_–N), nitrite nitrogen (NO_2_–N), ammonia nitrogen (NH_4_–N), and orthophosphate (PO_4_–P); the total phosphorus (TP) and the chemical oxygen demand (COD) were used as determinants of organic matter concentrations. The analyses followed the methods described in the Standard Methods (APHA 1998). In an attempt to devise a system to compare various parts of the Łyna River sections, we applied the Water Quality Index (WQI) in a modification of the original Scottish Water Quality Index (House 1989) and adapted by Cymes and Glińska-Lewczuk (2016) to low-gradient watercourses in northeastern Poland. The WQI, as one of the most widely used water quality procedures, allows for the determination of a potential economic use of water, which in itself is an important instrument to be employed in water management (dos Santos Simoes et al. 2008). The nine-parameter index is based on an aggregation of three groups of parameters: physical (temperature, conductivity, total suspended solids), chemical (dissolved oxygen pH, ammonia, nitrate), and organic (biological oxygen demand, chemical oxygen demand). All data were log_10_ transformed to standardize the data to a mean of 0 and a standard deviation of 1 to eliminate scale biases.

The microbial analysis of the water samples aimed to determine the following: heterotrophic plate count at 22 °C (HPC 22) such as autochthonous microorganisms, heterotrophic plate count at 37 °C (HPC 37), and fecal coliforms (FC) such as allochthonous microorganisms. Heterotrophic plate counts of bacteria (HPC 22, HPC 37) were determined in tryptic soy agar (TSA) (Oxoid) by plate count according to the Standard Methods (APHA 1998). To reduce the bacterial density, a decimal dilution series with PBS was prepared. Subsequently, 0.1 mL each of the well-homogenized solution (of the respective sample dilutions from 10^−1^ to 10^−4^) was placed on a TSA medium. Each sample was plated in duplicate and incubated for 24 h at 35 ± 2 °C (HPC 37) and 72 h at 22 ± 2 °C (HPC 22). Plates of those dilutions that yielded 30–300 colonies were chosen; the cultured colonies were counted and reported as colony forming units per milliliter of water (CFU mL^−1^).

To calculate the FC counts, the water samples were filtered using 0.45-mm pore size, 47-mm diameter sterile cellulose nitrate filters by “Sartorius.” Subsequently, the filters were placed on mFC agar (Difco) supplemented with 1 % rosolic acid, a selective medium specifically formulated for the enumeration of fecal coliforms by the membrane filtration method without prior enrichment according to the Standard Methods (APHA 1998). All the samples were incubated at 44.5 °C for 18–24 h. From each water sample, representative unique isolates, including a range of blue colonies, were randomly selected from the FC plates (Wohlsen 2011). Selected isolates, classified by this selection process as total thermotolerant fecal coliforms (FC), were enumerated and reported as the means of triplicate samples (total CFU 100 mL^−1^).

### Hydrological and meteorological data acquisition

Water flow data for the Łyna River during the observation period (hydrological year 2012) at Olsztyn (V), Smolajny (downstream of Dobre Miasto, IX), and Sępopol (XIV) have been provided by the IMWM in Poland. River flows for the stations X–XIII result from the calculations of IMWM data using the HEC-RAS software (HEC-RAS 2010). The software uses one-dimensional hydraulic analysis components for flow water surface profile computations.

The flows at stations I–IV were measured at the same time as the water sampling with an electromagnetic flow velocity sensor (Valeport, UK). The flow was calculated using the standard velocity–area method. Precipitation and related meteorological data for the region over the study period were measured at an automatic weather station network belonging to the University of Warmia and Mazury in Olsztyn, Poland.

### Data analysis and statistical methods

To assess the statistical differences among sampling locations for the water quality data and environmental data (seasons and flows), a non-parametric analysis of the variance Kruskal–Wallis (K–W) and Dunn’s tests as post hoc procedures (*P ≤* 0.05) was performed using the statistical software STATISTICA 10.0 PL. The Wilcoxon paired-sample test (Zar 1984) was used to compare paired water quality measurements upstream and downstream of each town.

The impact of environmental factors on the studied parameter concentrations in the analyzed water samples was determined by principal component analysis (PCA) with the use of the CANOCO 4.5 software package (ter Braak and Šmilauer 2002). PCA has allowed for the identification of the spatial (pollution from municipal origin) and temporal (seasons and flows) reduced factors affecting the quality of river water and the indication of polluting sources. The share of a given physico-chemical parameter in each season was graphically presented using a pie chart.

The varimax rotation method was used to apply factor analysis (FA). The main purpose of FA was to reduce the contribution of less significant variables and to further simplify the data structure that comes from PCA. The factor loading values were classified according to Liu et al. (2003) as “strong,” “moderate,” and “weak,” corresponding to absolute loading values of *>*0.75, 0.75–0.50, and 0.50–0.30, respectively.

Hierarchical cluster analysis (HCA) was used to investigate the grouping of the sampling sites along the Łyna River. Ward’s method using squared Euclidean distances was used as a measure of the similarity (Shrestha and Kazama 2007). The dendrogram was used to interpret the results of the cluster analysis. The spatial variability of water quality was determined from HCA using the linkage distance, reported as D_link_/D_max_. The quotient was then multiplied by 100 to standardize the linkage distance represented on the *x*-axis (Singh et al. 2005).

## Results and discussion

### Discontinuity in the longitudinal profile of the Łyna River

#### Physicochemical parameters

The water quality parameters varied markedly within and among the sampling stations along the Łyna River. The Łyna River water, like most of the other postglacial watercourses, is neutral to alkaline in nature and shows medium mineralization (ca. 600 μS cm^−1^) with dominating Ca^2+^ and HCO_3_^−^ ions in its overall chemical composition (Glińska-Lewczuk 2006). Based on hydrochemical profiling performed along the Łyna River, we have marked off two significant sections in a longitudinal profile of the river with a breaking point at Olsztyn—the biggest urban area in its catchment. The river upstream of Olsztyn represents natural values, not exposed to human activity, while the river downstream of Olsztyn is directly exposed to both urban and agricultural land use, which contributes to the extensive use of water and, therefore, its pollution. The river transit through the five towns (sampling stations between Olsztyn and Sępopol (VI–XIV) is represented by significantly higher values for the majority of the water quality parameters when compared to those in the upper river section (upstream of Olsztyn (I–V)).

DO showed large fluctuations with a clear decreasing trend in water aeration from 7.70 mg L^−1^ in the headwaters to 6.43 mg L^−1^ upstream of Olsztyn (Table 2). As expected, the DO concentrations decreased to less than 5.0 mg L^−1^ in the summer (not shown in the tables) at every station, but severe oxygen deficits only appeared at stations downstream of the cities, which could explain the stated variability. Consequently, in the “urban” section, the river water was characterized by low mean values of NO_3_–N (0.08–0.38 mg L^−1^) and relatively high values of NH_4_–N (0.15–0.62 mg L^−1^) (Table 2). An almost 4-fold increase in the mean NH_4_–N concentration was observed downstream of the WWTP in Olsztyn (VII) when compared to the Olsztyn center (VI). The concentrations of parameters such as NO_2_–N, NH_4_–N, TP, PO_4_–P, and COD were significantly lower at stations I–V than at stations VI–XIV (K–W test; *P* = 0.0001). However, DO and NO_3_–N showed opposite characteristics. They were significantly higher in the upper section than in the lower section (K–W test; *P* = 0.0001). A significant drawdown effect on dissolved oxygen in the river water beneath Olsztyn is likely to have ammonium oxidation and the decomposition of organic matter, which brings potentially harmful consequences for aerobic biota (Daniel et al. 2002). The high level of NH_4_–N concentration level likely stimulated the growth of nitrifying bacteria. Our results indicate that the pollution of the Łyna River with NH_4_–N downstream of Olsztyn or Lidzbark Warmiński facilitated the growth of nitrifying bacteria inoculated by the wastewater effluents. In general, the inorganic N species concentrations in the Łyna River were much less than 10 mg L^−1^, which was the US Environmental Protection Agency’s drinking water standard (US EPA 1986) or the EU’s directive for bathing (Directive 2006/7/EC).

**Table 2:** Physico-chemical parameters of the water along the Łyna River

Station no.	Location	pH	DO (mg L^−1^)		NH_4_–N (mg L^−1^)		NO_3_–N (mg L^−1^)		NO_2_–N (mg L^−1^)		TP (mg L^−1^)		PO_4_–P (mg L^−1^)		COD (mg L^−1^)		WQI-NSF	
		Median	Mean	±SD	Mean	±SD	Mean	±SD	Mean	±SD	Mean	±SD	Mean	±SD	Mean	±SD	WQI	Class
I	Headwaters	7.50	7.70b	1.81	0.05a	0.03	2.42d	0.27	0.010a	0.005	0.29a	0.17	0.08a	0.10	8.77a	5.75	81	II
II	Kurki	7.40	7.07	2.51	0.06a	0.05	0.94c	0.77	0.006a	0.002	0.38ab	0.20	0.12ab	0.07	17.64b	2.78	84	II
III	Ustrych	7.60	6.73	0.56	0.08a	0.07	0.47bc	0.14	0.006a	0.004	0.39ab	0.14	0.14ab	0.11	18.61b	4.38	84	II
IV	Ruś	7.40	6.46	1.91	0.08a	0.08	0.47bc	0.43	0.006a	0.001	0.40b	0.22	0.21b	0.08	19.14b	4.15	78	II
V	Upstream Olsztyn	7.30	6.43	1.03	0.14ab	0.15	0.27b	0.21	0.033b	0.046	0.41b	0.21	0.24b	0.06	19.51b	5.91	80	II
VI	Olsztyn center	7.50	5.64	0.82	0.15ab	0.54	0.11a	0.05	0.020	0.019	0.46bc	0.24	0.25b	0.07	21.56b	5.28	67	III
VII	Downstream of Olsztyn	7.20	5.39a	1.08	0.62d	0.69	0.08a	0.03	0.037b	0.028	0.76d	0.20	0.29bc	0.27	29.70c	7.86	48	IV
XIII	Upstream of Dobre Miasto	7.40	6.90	1.35	0.31b	0.12	0.21b	0.08	0.026	0.010	0.51c	0.29	0.28b	0.06	25.77bc	10.43	69	III
IX	Downstream of Dobre Miasto	7.40	6.01	0.92	0.44bc	0.48	0.12ab	0.05	0.024	0.011	0.51c	0.28	0.29b	0.09	26.63bc	14.48	65	III
X	Upstream of Lidzbark Warmiński	7.20	6.04	0.51	0.35b	0.22	0.21b	0.08	0.018	0.008	0.50c	0.23	0.28b	0.10	24.81c	6.18	61	III
XI	Downstream of Lidzbark Warmiński	7.50	5.87	0.70	0.59d	0.63	0.22b	0.11	0.034b	0.048	0.58c	0.25	0.28b	0.12	27.73c	9.70	54	III
XII	Upstream of Bartoszyce	7.30	5.84	0.60	0.22b	0.15	0.38b	0.18	0.032b	0.023	0.58cd	0.29	0.30bc	0.11	25.29c	5.53	54	III
XIII	Downstream of Bartoszyce = upstream of Sępopol	7.20	6.19	1.05	0.19b	0.13	0.24b	0.07	0.026	0.015	0.52c	0.14	0.35c	0.10	24.23c	4.45	51	III
XIV	Downstream of Sępopol	7.20	6.01	1.50	0.27b	0.24	0.11a	0.13	0.033b	0.019	0.54c	0.20	0.47d	0.12	27.31c	9.98	44	IV
I–XIV	Total	7.40	6.31	1.13	0.25	0.26	0.45	0.21	0.022	0.024	0.49	0.24	0.28	0.13	22.62	7.42	65	III

Values with the different lowercase letters denote sites significantly different in the Dunn’s test, a post hoc multiple comparisons procedure (*P* ≤ 0.05). WQI classess according to Cymes and Glińska-Lewczuk (2016)

Phosphates showed a gradually increasing pattern along the whole river length. Along the natural upper river region (I–V), the mean concentrations of PO_4_–P did not exceed 0.22 mg L^−1^ and 75 % of the samples contained less than 0.1 mg L^−1^, which is the concentration limit for streams recommended by US EPA (1986) to prevent excessive algal growth. In the urbanized river section, high concentrations of phosphate (P) (>0.1 mg L^−1^) were recorded in 100 % of the samples. The maximal values of phosphate (P) with a mean of 0.47 mg L^−1^ showed the samples taken downstream of Sępopol (at the Polish–Russian border). Downstream of the WWTP in Olsztyn (VII), both TP and COD showed significant increases (Dunn’s test, *P* ≤ 0.05) in their mean values to 0.76 and 29.70 mg L^−1^, respectively. In the longitudinal profile of the Łyna River, COD <20 mg L^−1^ was attributed to unpolluted river regions, while all the sampling stations along the urban regions exceeded that value.

Serial urban areas along the Łyna River have a deteriorating effect on the overall water quality, which is reflected by the WQI. All the sampling stations along the unpolluted stretch of the Łyna River are characterized by very good quality WQI ratings, with mean scores >80 points (Table 2). Stations located downstream of the towns had significantly lower WQI values (44–65) when compared to the stations located upstream of the towns (51–80). Except for the station located downstream of the WWTPs in Olsztyn (WQI = 48) and Sępopol (WQI = 44), no bad rating for water quality was recorded. The differences in the WQI ratings showed that the Łyna River can be divided into two sections composed of stations I–V (class II) and stations VI–XIV (classes III–IV). It has been reported (Heidenwag et al. 2001; Vaikasas and Dumbrauskas 2010) that rivers with slopes <1 ‰ and thus lower velocities seem to have higher potential for self-purification, as they maintain the natural retention threshold for pollutants. The evidence for the self-purification potential of the Łyna River within its “urban section” might be a gradual decrease in the nutrient concentrations between the towns, which resulted from small slopes of the river channel (0.12–1.46 ‰). Within a distance of 38 km between Olsztyn (VII) and Dobre Miasto (VIII), with no lateral input from point sources, the concentrations of NH_4_–N decreased by 50 %, PO_4_–P by 40 %, and NO_2_–N and TP by 30 %.

This overall pattern for the physico-chemical parameters used in this study indicates significant contributions from the most populated towns toward the deterioration of river water quality, in spite of the modern WWTPs (Table 3). Two of the five towns located on the Łyna River significantly changed the river water quality, namely, Olsztyn and Lidzbark Warmiński (Wilcoxon paired-test, *P* = 0.0001).

**Table 3:** Wilcoxon paired-test between the overall water quality in the samples taken from (A) natural sections and (B) urbanized sections of the Łyna River upstream and downstream of the towns along the Łyna River

Location along the Łyna River		Sampling stations compared		*N*	*T*	*Z*	*P* value
(A) Natural section		Headwaters (I)	Ruś (IV)	96	888	2.39	0.017*
		Headwaters (I)	Olsztyn (VI)	96	870	2.64	0.008**
(B) Urbanized section	Olsztyn	Upstream (V)	Downstream (VII)	96	704	3.93	0.000***
	Dobre Miasto	Upstream (VIII)	Downstream (IX)	94	1293	0.70	0.484
	Lidzbark Warmiński	Upstream (X)	Downstream (XI)	92	548	4.41	0.000***
	Bartoszyce	Upstream (XII)	Downstream (XIII)	94	1182	1.45	0.146
	Sępopol	Upstream (XIII)	Downstream (XIV)	96	963	1.81	0.071

*N* number of samples, *T* test statistics, *Z* normal approximation

*P ≤ 0.05; **P ≤ 0.01; ***P ≤ 0.001

#### Microbiological parameters

**Fig. 2:**
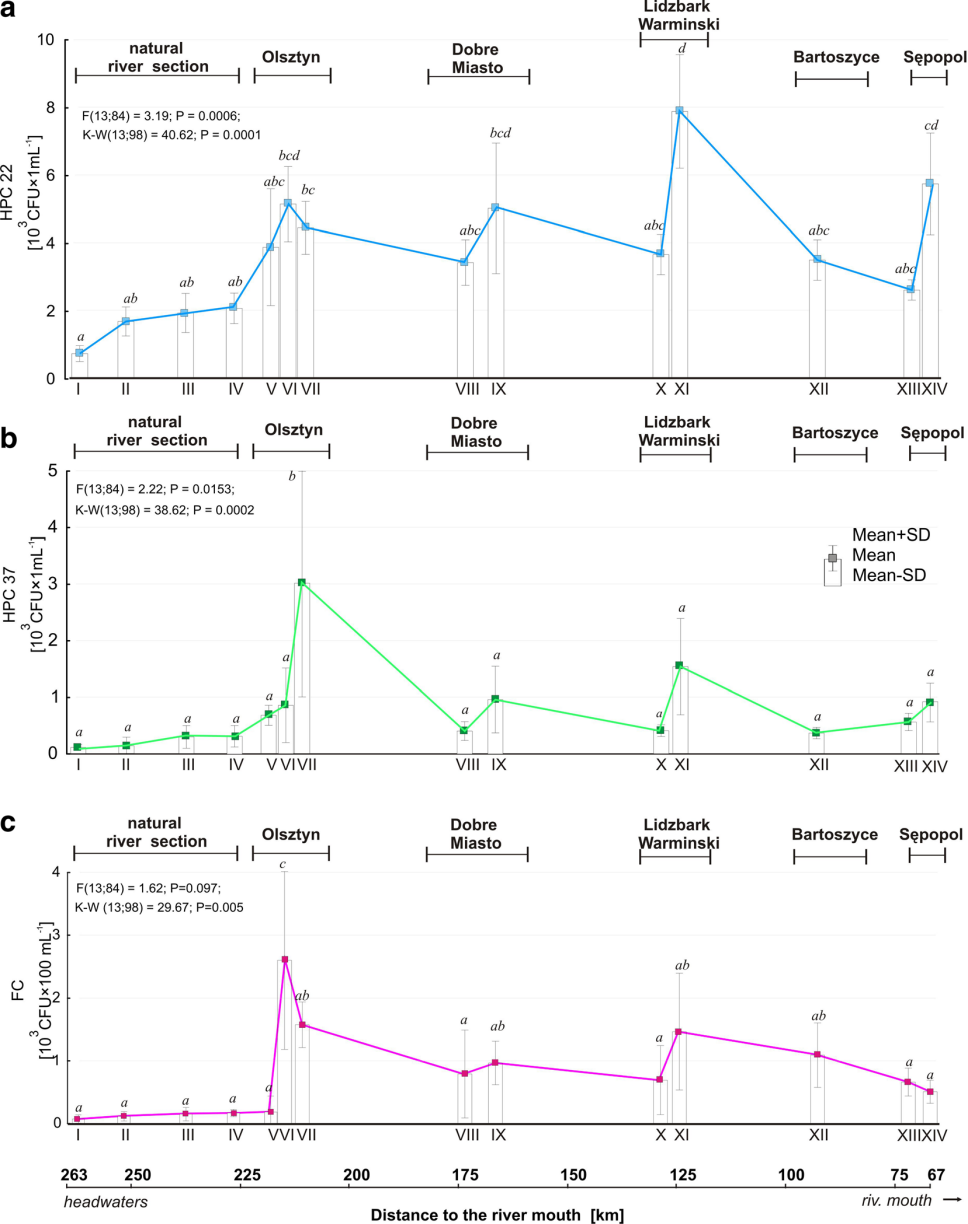
Longitudinal profiles of the microbiological parameters (mean ± SD) of the water in the Łyna River at sampling sites *I*–*XIV*. **a** HPC 22, **b** HPC 37, and **c** fecal coliforms (FC). *Different letters* denote groups of means that are significantly different in the non-parametric Dunn’s test at *P* ≤ 0.05

The bacterial colonies at stations I to IV formed a deeply branching group (Fig. 2), reflecting a differentiation in the physico-chemical parameters along the longitudinal dimension of the Łyna River. In the river’s upper region, comparing the HPC 22 in water from the first sampling station (I) with that from subsequent stations (II–IV) showed its relationship with increasing distance from the headwaters (Fig. 2a). The mean bacteria counts from this group ranged from less than 0.1 × 10^3^ CFU mL^−1^ to less than 2 × 10^3^ CFU mL^−1^ and showed no significant difference among the stations (K–W test; *p* = 0.0001). In the urbanized regions, the mean counts of HPC 22 ranged from 2.4 × 10^3^ CFU mL^−1^ at Sępopol to 7.98 × 10^3^ CFU mL^−1^ at Lidzbark Warmiński. In general, these bacteria were less numerous in water samples collected upstream than downstream of the towns; however, only the examples from Lidzbark Warmiński and Sępopol showed statistical significance (K–W and post hoc Dunn’s tests, *P* ≤ 0.05).

The HPC 37 differed by several orders of magnitude with regard to the section, sampling station, and season. Throughout the entire study period, the lowest HPC 37 values were noted in water samples collected from sampling stations I–IV. They ranged from 0 CFU mL^−1^ in the headwaters (I) to 0.4 × 10^3^ CFU mL^−1^ at Ustrych (III) and Ruś (IV). Higher bacterial concentrations were found in the water samples collected along the urban section of the river, in particular, at the stations located downstream of the towns (VI, VII, IX, XI, XII, and XIV). The mean counts of HPC 37 ranged from 0.3 × 10^3^ CFU mL^−1^ upstream of Lidzbark Warmiński (X) to 3 × 10^3^ CFU mL^−1^ downstream of Olsztyn (VII). HPC 22 showed a similar pattern to HPC 37 along the river profile, with no significant differences between the stations (Dunn’s test, *p* ≤ 0.05) except for station VII (downstream of Olsztyn).

During the recording period, FC did not pose a health risk along the upper section of the Łyna River (FC <1 CFU 100 mL^−1^). However, FC was progressively increasing in numbers down the river course. An abrupt increase in FC reached 2.5 × 10^3^ CFU 100 mL^−1^ at station VI, where the river receives contaminants from diffuse sources along the town of Olsztyn.

In general, the riverine bacteria developed semi-gradually over the course of the river. This pattern was disrupted in the urban regions (VII–XIV) by an abrupt increase in apparent bacterial richness and a clear shift in the majority of the nutrients. The input of treated sewage altered the investigated parameters of the water. Our data indicate that the intrinsic factors determining bacterial abundance are man-made pollutants received by the river from urban areas with effluents from wastewater treatment plants. In particular, the sampling station at the Olsztyn center (VI) marks the turning point with the highest average concentrations of apparent bacteria. The VI sampling point is located at a city park, a popular site for recreation for the citizens and tourists. Some authors (e.g., Korzeniewska 2005, Edge and Hill 2007, Wright et al. 2009; Kacar 2011; Daly et al. 2013; Garcıa-Armisen et al. 2014; Vincy et al. 2015) reported that the contamination of public recreational places with fecal bacteria was mainly caused by pet animals’ feces. In this study, only the samples collected at stations I–IV were within the contamination limits recommended by the Bathing Water Directive (2006/7/EC) for recreational waters (<0.5 × 10^3^ CFU 100 mL^−1^). In line with the criteria set out by the Directive, all the water samples collected in the urban sections failed to meet the minimal requirements and were classified in the “poor quality” category.

### Multivariate analyses of the water quality

**Fig. 3:**
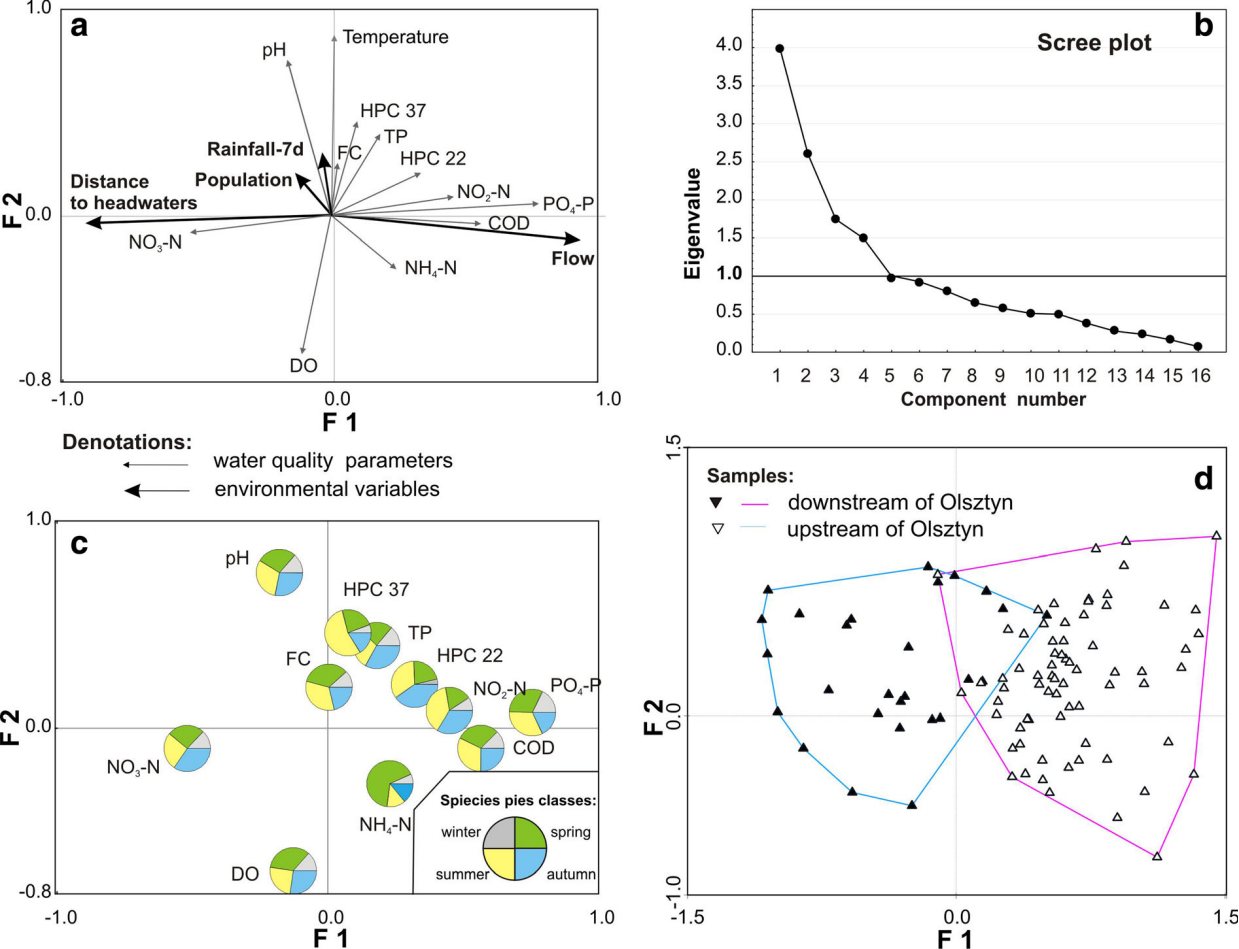
Results of principal component analysis performed with the environmental variables and water quality data along the Łyna River (*P* ≤ 0.05). **a** *Biplot* of significant environmental variables and water quality data related to factor 1 (*F1*) and factor 2 (*F2*). **b** *Scree plot* for eigenvalues. **c** *Pie charts* showing the share of each parameter values in each season. **d** Plot of the samples’ distribution. Two distinct groups of samples have been selected: *blue area with black triangles* is related to the unpolluted river section, and *red area with white triangles* denotes the anthropogenically polluted river section

Factor analysis (FA) and principal component analysis (PCA) were accomplished on the standardized data sets for the 14 sampling sites. Factor analysis of the data set outputs a four-component model with 75.36 % of the total variance explained (Fig. 3a; Table 4). The significance of any factor was determined by an eigenvalues >1.0 (Fig. 3b). The first component (F1) describes 31 % of the total variance and includes strong loadings (>0.75) of PO_4_–P and environmental variables with distance to the headwater and flow (Table 4). COD and NO_2_–N are also related to F1, but they are noted by moderate loadings. Both parameters have positive relations with the number of bacteria colonies, especially HPC 22. The values of COD and NO_2_–N increased from the upstream to downstream stations of the Łyna River (Fig. 3a), indicating that water pollution associated with domestic, municipal, and land use activities. This was largely expected because despite the connections of households to WWTPs, the domestic effluents of unsewered areas and surface runoff from the large, impervious urban areas of Olsztyn and Lidzbark Warmiński discharge their wastewater with rainfall directly into the Łyna River. Thus, heavy rainfalls can be attributed to the increase in FC from non-point sources along, e.g., the Olsztyn center. However, this factor shows a strong relation to the fourth component (F4).

**Table 4:** The factor loadings after the varimax rotation of the water quality data

Parameters	F1	F2	F3	F4
Distance to headwaters (km)	***–*** 0.93***	–0.04	0.06	0.03
Population	–0.24	0.09	0.83***	–0.08
Flow	0.92***	–0.10	–0.06	–0.05
Temperature	0.00	0.88***	–0.04	0.06
Rainfall (7-day sum)	–0.03	0.27	0.02	0.86***
HPC 22	0.33*	0.21	0.43*	0.01
HPC 37	0.09	0.46*	0.39*	–0.22
FC	0.01	0.21	0.43*	0.40*
pH	–0.18	0.76***	–0.08	0.37*
COD	0.55**	–0.07	0.49*	–0.15
DO	–0.13	–0.68**	–0.16	0.17
PO_4_–P	0.76***	0.06	0.15	–0.10
TP	0.17	0.40*	0.23	–0.66**
NH_4_–N	0.23	–0.25	0.70***	0.01
NO_2_–N	0.44*	0.09	0.31*	0.18
NO_3_–N	–0.52**	–0.09	–0.47*	–0.05
% Variance	31.01	18.29	15.94	10.12
% Of cumulative variance	31.01	49.30	65.24	75.36

Values in italics indicate statistically significant scores

*0.30–0.50 (weak); **0.50–0.75 (moderate); ***>0.75 (strong loading)

Factor 2 shows approximately 18.29 % of the total variance (Table 4). It has two parameters with strong positive loadings (pH and temperature) and one with moderate negative loading (DO). The loadings of TP and HPC 37 increase with the temperature and preceding rainfalls. Factor 3 shows approximately 16 % of the total variance and has only two parameters with strong significant loadings, which are NH_4_–N and the number of inhabitants, while HPC 22, HPC 37, FC, COD, and NO_2_–N showed weak loadings; thus, this factor attributes pollution to domestic wastewater. Factor 4, with 10 % of the variance, is significantly related to the rainfall sum, which has an important influence on FC counts and the inverse loading of TP.

A trend toward higher apparent FC bacterial richness is likely caused by man-made factors rather than environmental factors that partially confirmed the results of Kacar (2011), who found no significant relationships between the fecal indicator bacteria and environmental factors. We argue that point source contamination tends to be directly discharged into river water, generating contamination variability that is often unrelated to runoff variability and meteorological factors. Conversely, diffuse contamination requires transport by means of runoff into surface water (St Laurent and Mazumder 2014). Therefore, the variability in fecal contamination may be more strongly associated with hydrometeorological conditions in watersheds, where contamination is associated with diffuse sources. This can be observed along the city of Olsztyn (see station VI), where the highest FC concentrations come from the impervious urban area washed out by heavy rainfalls, while the correlation between FC counts and rainfall sums within the past 7 days was significant and amounted to *r* = 0.36 (*P* ≤ 0.05, not shown in tables). Please also consider the lack of a relation between FC and COD (Fig. 3a). Based on the obtained results, we may conclude that HPC 37 is more related to point sources, herein effluents from WWTPs, than FC, as the example of Olsztyn shows, to the direct diffuse input of contaminants into the river.

The highest counts of HPC 22 were recorded in autumn and spring, whereas the highest counts of HPC 37 occurred mainly during spring and summer (data not shown). This observation might be assigned to higher levels of nutrients, particularly NH_4_–N, PO_4_–P, and COD (Fig. 3c), which was also reported by Kacar (2011) for Turkish rivers and for other postglacial rivers in Poland by Gołaś et al. (2008) or Donderski and Wilk (2002). In addition, no clear FC-dominating season can be observed in the pie charts (Fig. 3c).

**Fig. 4:**
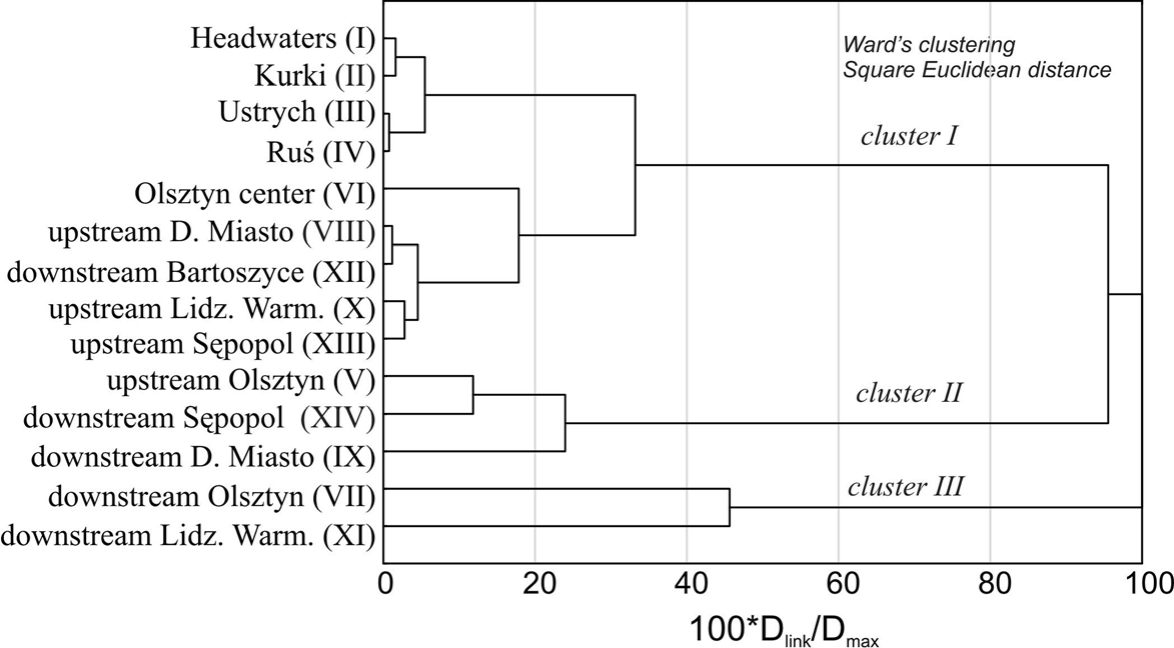
Dendrogram of hierarchical cluster analysis (HCA) for 14 sampling sites along the Łyna River based on the physico-chemical and bacteriological parameters of the water. The clusters are statistically significant when D_link_/D_max_ ∗ 100 < 50

By looking at the patterns of the water quality parameters along the Łyna longitudinal profile, it becomes possible to compare the 14 sampling stations with each other and visualize their relationships in the form of a dendrogram (Fig. 4). Cluster I was identified as a group consisting of stations I to IV (Headwaters → Ruś) and the stations located at Olsztyn’s center (VI) and downstream of the towns (VIII, XII, X, XIII). In cluster I, the group of stations corresponds to the least polluted river water. Within cluster II, the sites located at upstream Olsztyn (V), downstream Sępopol (XIV), and downstream Dobre Miasto (IX) were the most similar to each other. The inclusion of the sampling station farthest downstream (downstream Sępopol) in cluster II suggests the self-purification and assimilative capacity of the river. Cluster III formed the group consisting of the most polluted sites: downstream Olsztyn (VII) and Lidzbark Warmiński (XI). These stations receive pollution mostly from domestic wastewater and WWTP effluents located in the city areas (Fig. 4).

In upper reach of the river (headwater area), a portion of nutrients removed from the water column is removed permanently via denitrification, but the majority are only delayed temporarily, e.g., via assimilatory uptake (Mulholland et al. 2008), and are eventually available for export downstream the river. Comparative analyses of water quality change along natural (upstream) and urban (downstream) sections of the Łyna River have led us to conclusions supporting the outcomes of the study performed by Seitzinger et al. (2002) and Reisinger et al. (2015) that the nutrient drawdown in the downstream river section is more efficient at removing nutrients per unit stream length compared to the headwater section.

Our results showed that the relative influences of five urban areas and natural environmental factors on the ecosystem of one river showed that urbanization was most likely the primary determinant of river water quality deterioration. The established relationships between five consecutive towns and hydrochemical and microbiological conditions, however, did not indicate a specific threshold of effects but showed that the spatial distribution of impervious surfaces and sanitary infrastructure has a direct impact on the longitudinal changes in water quality parameters of the Łyna River.

Due to spatial heterogeneity of land use, monitoring of the changes in water quality, retention, and export of pollutants along the urban river continuum is critical for predicting downstream alterations of ecosystem functions, managing pollutant exports, and finally assessing the needs of restoration activities at a scale river of the river as its watershed.

## Conclusions

Our study investigated the influence of serial urban areas on the physico-chemical water quality parameters and bacterial community along the Łyna River in northern Poland, a representative for low-gradient postglacial watercourses in the temperate climatic zone. The hydrochemical and microbiological profiling of the Łyna River established two river sections with different levels of water quality: (1) the upper, unpolluted section from the headwaters to Olsztyn and (2) the lower section with anthropogenic influences, mostly from urban areas. In this study, we demonstrated that the water quality in the upper region, in terms of both the physico-chemical and microbiological parameters, may be considered as a benchmark of the geochemical background for the assessment of water chemistry conditions in the context of the entire region.

Abrupt changes in the physico-chemical and microbiological parameters of the water have been found downstream of the five towns that the Łyna River passes. The spatial and temporal variations of the quality and microbiology of the river water is the hallmark of urban pollution. The application of PCA and cluster analysis allowed for the grouping of river water sampling stations based on the seasonal and spatial criteria. The rotated principal components have demonstrated that (i) the nutrient contents in the upper region are more seasonal and climate dependent, thus pointing to their natural origin (geochemical background) and that (ii) pollution from organic matter, nutrients, and microorganisms originates from anthropogenic sources, mainly as municipal wastewater, which is more resistant to hydrology and seasonal variations.

Our conclusion is that cities are no longer isolated elements in the landscape but are parts of hydrologically connected ecosystems that encompass large areas of the landscape into one “urban river continuum.” The present-day management of an urban river continuum requires investigating the cumulative and/or overlapping effects of all urban areas as pollutant sources and the alternating fluxes and flow paths of materials and energy into the receiving water downstream. Considering the river as a medium that transfers a variety of pollutants, understanding the impacts of a single urban area will be equally as important as the urban river continuum in predicting the impacts of man-made pollutants on river water quality.

The quantitative characteristics of the physico-chemical parameters and the abundance of bacteria along a longitudinal profile of a river obtained in the research procedure have made it possible to define a base for the factors underlying the functioning of the Łyna River as a present-day regularity in postglacial areas situated in the temperate climatic zone.
